# SATB2, CKAE1/AE3, and synaptophysin as a sensitive immunohistochemical panel for the detection of lymph node metastases of Merkel cell carcinoma

**DOI:** 10.1007/s00428-023-03691-7

**Published:** 2023-12-08

**Authors:** Anna Szumera-Cieckiewicz, Daniela Massi, Angelo Cassisa, Mateusz Krzyzinski, Monika Dudzisz-Sledz, Przemyslaw Biecek, Piotr Rutkowski, Andrzej Marszalek, Mai P. Hoang, Piotr Donizy

**Affiliations:** 1 Department of Pathology, Maria Sklodowska-Curie National Research Institute of Oncology, W.K. Roentgena 5, 02-781 Warsaw, Poland; 2 Member of EORTC Melanoma Pathology Working Group, Brussels, Belgium; 3 Section of Pathological Anatomy, Department of Health Sciences, University of Florence, Florence, Italy; 4 Section of Pathology, Department of Oncology, San Giovanni Di Dio Hospital, USL Centro Toscana, Florence, Italy; 5 Faculty of Mathematics and Information Science, Warsaw University of Technology, Warsaw, Poland; 6 Department of Soft Tissue/Bone Sarcoma and Melanoma, Maria Sklodowska-Curie National Research Institute of Oncology, Warsaw, Poland; 7 Department of Pathology, Poznan University Medical Sciences and Greater Poland Cancer Center, Poznan, Poland; 8 Department of Pathology, Massachusetts General Hospital and Harvard Medical School, Boston, USA; 9 Department of Clinical and Experimental Pathology, Wroclaw Medical University, Borowska 213, 50-556 Wroclaw, Poland; 10 Department of Pathology and Clinical Cytology, Jan Mikulicz-Radecki University Hospital, Wroclaw, Poland

**Keywords:** Merkel cell carcinoma, Lymph node metastases, Immunohistochemistry

## Abstract

Histopathological evaluation of lymph nodes in Merkel cell carcinoma has become crucial in progression estimation and treatment modification. This study was undertaken to determine the most sensitive immunohistochemical panel for detecting MCC nodal metastases. We included 56 patients with 102 metastatic MCC lymph nodes, which were tested with seven antibodies: cytokeratin (CKAE1/AE3), CK20, chromogranin A, synaptophysin, INSM1, SATB2, and neurofilament (NF). Tissue microarrays (TMA) composed of 2-mm tissue cores from each nodal metastasis were constructed. A semiquantitative 5-tier scoring system (0%, < 25%, 25–74%, 75–99%, 100% positive MCC cells with moderate to strong reactivity) was implemented. In the statistical assessment, we included Merkel cell polyomavirus (MCPyV) status and expression heterogeneity between lymph nodes from one patient. A cumulative percentage of moderate to strong expression ≥ 75% of tumoral cells was observed for single cell markers as follows: 91/102 (89.2%) SATB2, 85/102 (83%) CKAE1/AE3, 80/102 (78.4%) synaptophysin, 75/102 (75.5%) INSM1, 68/102 (66.7%) chromogranin A, 60/102 cases (58.8%) CK20, and 0/102 (0%) NF. Three markers presented a complete lack of immunoreactivity: 8/102 (7.8%) CK20, 7/102 (6.9%) chromogranin A, and 6/102 (5.9%) NF. All markers showed expression heterogeneity in lymph nodes from one patient; however, the most homogenous was INSM1. The probability of detecting nodal MCC metastases was the highest while using SATB2 as a first-line marker (89.2%) with subsequential adding CKAE1/AE3 (99%); these results were independent of MCPyV status. Synaptophysin showed a superior significance in confirming the neuroendocrine origin of metastatic cells. This comprehensive analysis allows us to recommend simultaneous evaluation of SATB2, CKAE1/AE3, and synaptophysin in the routine pathologic MCC lymph node protocol.

**Supplementary Information:**

The online version contains supplementary material available at 10.1007/s00428-023-03691-7.

## Introduction

Merkel cell carcinoma (MCC) is a rare, aggressive primary cutaneous neuroendocrine carcinoma with a high propensity for lymphatic spread [1]. Correct histopathologic detection of nodal MCC metastases is crucial because involvement of lymph nodes at diagnosis is significantly associated with a worse outcome [2, 3]. According to the 8th edition of AJCC classification, any isolated tumor cells in a lymph node are classified as micrometastases (pN1a). However, there is no well-established standardized protocol of pathologic examination with a description of which immunohistochemical (IHC) markers should be used in routine specimens from sentinel lymph node biopsies (SLNB) and/or lymphadenectomies (LNB).

SLNB is a widely used method for pathologic staging as the guide for possible further radical lymphadenectomy [4]. Although extensive MCC metastases in a lymph node are easily detected in hematoxylin and eosin (HE) stained sections, single cells and small foci of micrometastatic MCC are exceedingly difficult to identify in the background of lymphoid cells. The use of selected immunostains has been shown to increase the sensitivity of identifying occult lymph node metastases. Still, these studies are based only on single immunostains and/or a relatively small number of cases [5, 6].

In recent years, new MCC markers were reported, such as INSM1 (insulinoma-associated protein 1) and SATB2 (special AT-rich sequence-binding protein 2), but the utility of these markers in a routine evaluation of MCC nodal metastases has not yet been investigated [7–10].

We herein aimed to compare immunohistochemical markers commonly used for detecting metastatic MCC. The analyzed panel included 7 antibodies: cytokeratin (clone CKAE1/AE3), cytokeratin 20 (CK20), chromogranin A (ChgA), synaptophysin (Syn), neurofilament (NF), INSM1, and SATB2. Since the single metastatic MCC cell changes the staging, we implemented strict but applicable to routine practice scoring cut-offs. To the best of our knowledge, this study is the first to comprehensively describe the diagnostic utility of a wide panel of IHC markers in a large cohort of metastatic nodal MCC cases.

## Material and methods

### Patients

In total, 102 lymph nodes with MCC metastases were included in this study from 5 clinical institutions in Poland, Italy, and the USA. The study was approved by the Ethics Committee of the Maria Sklodowska-Curie National Research Institute of Oncology, Warsaw, Poland (No. 9/2021). The diagnoses of metastatic MCC were histopathologically confirmed by the contributing pathologists and the first and corresponding authors (ASC and PD). Lymph nodes with extensive metastases were included (≥ 0.5 cm) after quality control analysis (absence of necrosis, validated preanalytical process, and paraffin blocks within 10 years from diagnosis).

### Immunohistochemistry

Tissue microarrays (TMA) composed of 2-mm tissue cores from each nodal metastasis were constructed. Immunohistochemical studies were performed on 4-μm-thick tissue sections using an automated Autostainer Link 48 (DAKO, Santa Clara, CA, USA) with primary antibodies against SATB2 (SATBA4B10, 1:100, Zytomed Systems, Berlin, Germany), cytokeratin (AE1/AE3, prediluted, DAKO, Santa Clara, CA, USA), synaptophysin (DAK-SYNAP, prediluted, DAKO, Santa Clara, CA, USA), INSM1 (BSB-123, dilution 1:150, Bio SB), chromogranin A (DAK-A3, prediluted, DAKO, Santa Clara, CA, USA), CK20 (Ks 20.8, prediluted, DAKO, Santa Clara, CA, USA), and NF (2F11, prediluted, DAKO, Santa Clara, CA, USA) for 102 metastatic MCC lymph nodes. In all cases, we also evaluated Merkel cell polyomavirus (MCPyV) status by expressing MCPyV large T antigen (CM2B4, 1:200; Santa Cruz Biotechnology, Santa Cruz, CA, USA). For 10% of randomly selected cases, whole slides were also stained. Normal human tissues were included in 4 TMAs (8 reactive tonsils, 4 normal testes, 4 livers, and 4 kidneys with no pathologic changes) (Supplemental Fig. 1).

The modified 5-tier scoring system with cut-offs, as previously reported, was used for all analyzed markers: 0, 0%; 1, < 25%; 2, 25–74%; 3, 75–99%; 4, 100% of MCC cells with moderate to strong reactivity [11]. Tumor cells with positive CM2B4 nuclear staining of any intensity were considered positive [12].

All immunohistochemically stained slides were evaluated by two independent pathologists (ASC and PD) who were blinded to the clinical data, and discordant cases were reviewed together until a consensus was reached.

### Statistical analysis

Statistical analysis was performed using the R language [13]. The visual analyses were created using the ggplot2 package [14]. The Leti index was used to quantify the inter-patient heterogeneity [15]. To test the correlation between the analyzed markers, Spearman’s rank correlation coefficient was used. A *p*-value below 0.05 was considered significant for all comparisons. To find the most efficient order of testing a selected set of markers, we establish rules presented in decision trees, which show the probability of detection (sensitivity) achieved for a given set of proteins and its changes with the addition of the next ones.

## Results

### Patients

The study group consisted of 56 patients (32 males and 24 females; mean age 71, range 38–87 years) with extensive (≥ 0.5 cm) nodal MCC metastases. In total, 102 metastatic lymph nodes were included.

### Distribution of IHC markers

A cumulative percentage of moderate to strong expression in ≥ 75% of tumoral cells was observed for single cell markers as below: 91/102 (89.2%) SATB2, 85/102 (83%) CKAE1/AE3, 80/102 (78.4%) synaptophysin, 75/102 (75.5%) INSM1, 68/102 (66.7%) chromogranin A, 60/102 cases (58.8%) CK20, and 0/102 (0%) NF. On the contrary, three markers presented a complete lack of immunoreactivity: 8/102 (7.8%) CK20, 7/102 (6.9%) chromogranin A, and 6/102 (5.9%) NF. A detailed frequency description of all evaluated markers is presented in Table 1 and Supplemental Fig. 2.

**Table 1:** Distribution of immunohistochemical expression of separate markers

IHC marker	0: 0%	1: < 25%	2: 25–74%	3: 75–99%	4: 100%
SATB2	0 (0%)	6 (5.88%)	5 (4.9%)	39 (38.24%)	52 (50.98%)
CKAE1/AE3	0 (0%)	7 (6.86%)	10 (9.8%)	53 (51.96%)	32 (31.27%)
Syn	0 (0%)	8 (7.84%)	19 (18.63%)	63 (61.76%)	17 (16.67%)
INSM1	0 (0%)	8 (7.84%)	19 (18.63%)	75 (73.53%)	0 (0%)
ChgA	7 (6.86%)	11 (10.78%)	16 (15.69%)	40 (39.22%)	28 (27.45%)
CK20	8 (7.84%)	23 (22.55%)	11 (10.78%)	55 (53.92%)	5 (4.9%)
NF	6 (5.88%)	79 (77.45%)	17 (16.67%)	0 (0%)	0 (0%)

**Fig. 1:**
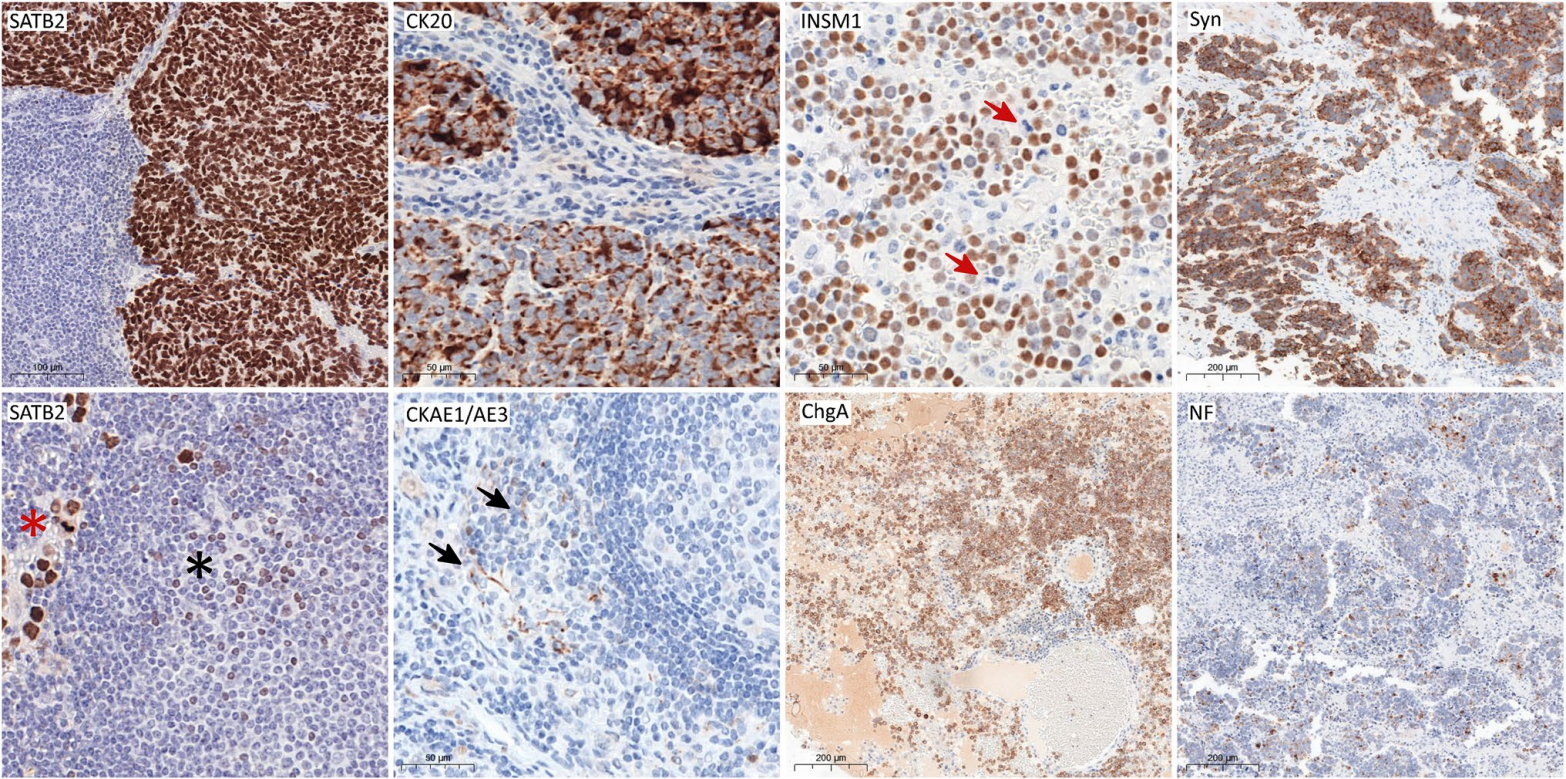
Immunohistochemical characteristics of analyzed markers. SATB2 expression profile: most of the cases showed a homogenous, strong, nuclear reaction in 100% of MCC cells, the weak to moderate expression was seen in lymphocytes (mostly in germinal centers; black asterix), but the cell morphology and quantity of the stain are helpful in indicating the MCC metastasis (red asterisk); CK20 distribution included submembranous and dot-like reactions; in lymph nodes, some dendritic cells (arrow heads) may present positive CKAE1/AE3 stain and it could be misinterpreted with MCC metastases (black arrows) especially when the only pattern of expression is dot-like; INSM1 distribution pattern was nuclear, but no cases showed 100% positivity because the cells with mitotic figures were always negative (red arrow); synaptophysin presented a mostly high percentage of positive stain; in a majority of cases, the groups of weakly positive or negative cells were seen, but there were no entirely negative cases; chromogranin A was expressed primarily on below 75% of cells with submembranous and cytoplasmic stain, focally significant unspecified background is observed; NF expression was very low—the dot-like expression in majority of cases (> 70% of cases) was below 25% of cells

SATB2 were characterized by uniform strong nuclear expression; worth noticing some reactive lymphocytes presented weak to moderate SATB2 staining but the difference in size and shape is helpful in the distinction. We found that nodal dendritic cells were focally CKAE1/AE3-positive with weak to moderate intensity. A surprising observation was that there were no cases with 100% INSM1-positive immunoreactivity due to the lack of INSM1 expression in actively dividing tumoral cells (mitoses). CK20 presented both cytoplasmic and dot-like patterns. For NF, dot-like perinuclear localization was a dominant expression pattern. The typical expression of IHC with pitfalls is shown in Fig. 1.

**Fig. 2:**
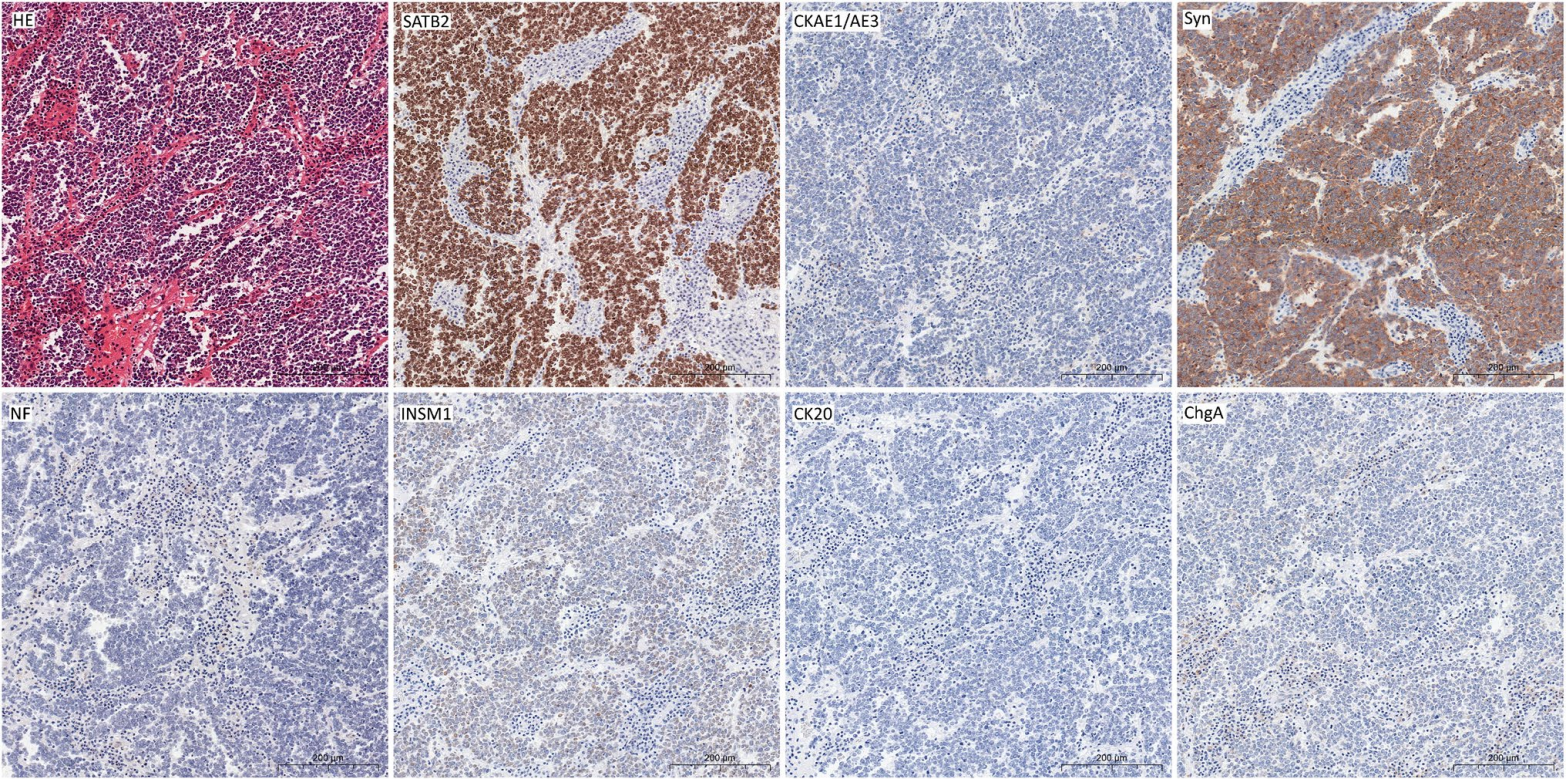
CK20 negative case showed high SATB2 and synaptophysin expression; CKAE1/AE3, INSM1, chromogranin, and NF were detected in single cells (below 25% of cells)

**Fig. 3:**
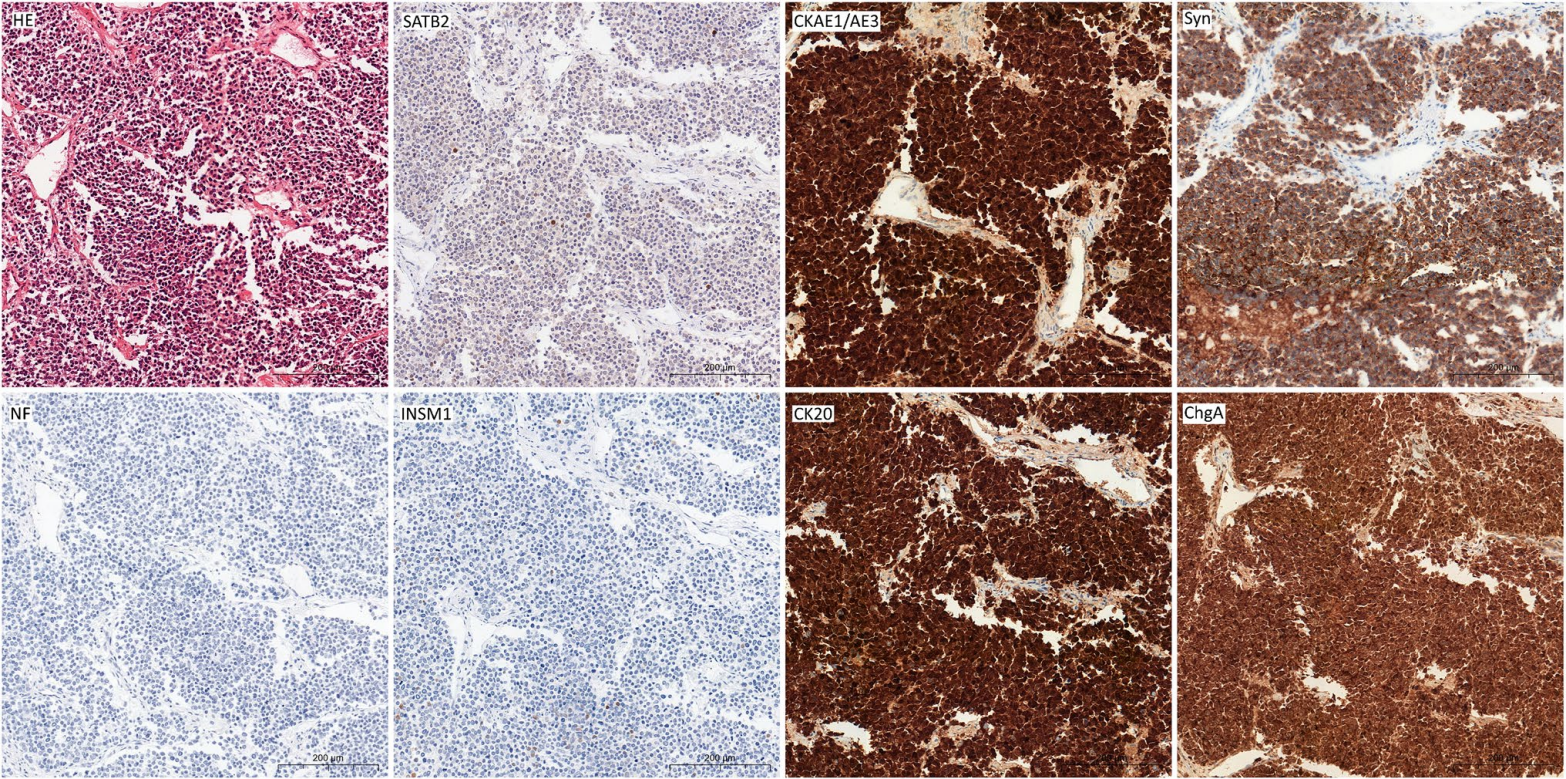
Case with low SATB2 expression (below 25% cells) was uniformly positive with CKAE1/AE3, CK20, synaptophysin, and chromogranin A; INSM1 was identified in single cells; NF was negative

Eight CK20-negative nodal metastases were positive for SATB2 (7 cases with moderate to strong expression in ≥ 75% of metastatic cells; 1 case with moderate to strong expression in less than 25% of tumoral cells) (Fig. 2). Six cases with SATB2 cut-off < 25% presented CKAE1/AE3 and/or synaptophysin moderate to strong expression in ≥ 75% (Fig. 3).

### Correlation of IHC and proposal of IHC panel

Statistical analysis revealed mild to moderate linear correlations between most analyzed markers (Supplemental Fig. 3). Interestingly, our cohort showed a complete lack of relationship between SATB2 and CKAE1/AE3 immunoreactivities (Spearman’s correlation = 0.03, *p* = 0.75), which indicated their possible additive effect on the detection of MCC metastases. 10/11 (91%) cases with SATB2-low manifested CKAE1/AE3-high expression (Supplemental Fig. 3).

**Fig. 4:**
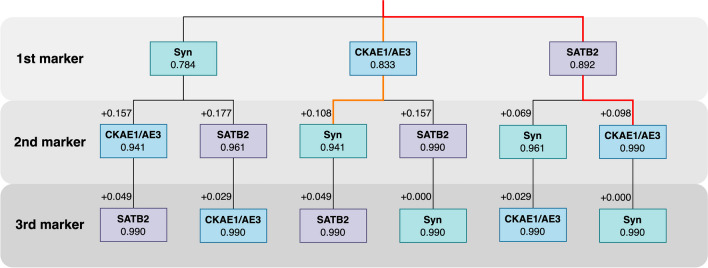
The decision tree shows the sensitivity of sets of markers included in the proposed panel. Scoring 3 and 4 (≥ 75% of MCC cells with moderate to strong reactivity) was used as an indicator of the detection of lymph node metastases. At the first level of the tree, one marker is selected, and the achieved sensitivity is determined (as a fraction of 102 analyzed lymph nodes in which metastases were detected based on the analysis of the selected marker and the adopted definition of the detection). Then, the next marker is selected from among the two remaining from the previous step. Sensitivity is determined by the number of nodes appropriately classified with at least one marker. In addition, the sensitivity change is shown on edge. The third level is analogous to the second one, but only one of the remaining markers can be selected. The path marking the optimal testing order, assuming the availability of all markers, is marked in red. The orange color represents the optimal ordering when SATB2 is not available

The most sensitive set of all analyzed IHC markers for detecting nodal MCC metastases were SATB2, CKAE1/AE3, and synaptophysin. Our cohort of patients had no negative cases for these 3 markers. When we used SATB2 as a first-line IHC marker, the probability of detecting nodal MCC metastases was 89.2%. If we subsequently performed IHC for CKAE1/AE3, the probability of detecting nodal MCC metastases was 99% (Fig. 4). Adding synaptophysin was recommended for the confirmation of the neuroendocrine feature of analyzed metastatic cells. In the absence of SATB2, a combination of CKAE1/AE3 and synaptophysin showed a 94.1% probability of detecting nodal MCC metastases.

### Heterogeneity of single IHC markers

We observed different levels of intrapatient heterogeneity of IHC stains in cases with more than one metastatic lymph node (Supplemental Figs. 4 and 5). Almost all markers showed a lack of pure homogeneity of their expression in lymph nodes from one patient. SATB2, chromogranin A, and synaptophysin were the markers with the widest spectrum of different percentages of positive tumoral cells in separate metastatic lymph nodes from the same patient. The most homogenous marker was INSM1 (Supplemental Figs. 4 and 5). Nevertheless, SATB2 and synaptophysin detected the highest number of tumoral cells in one patient.

### MCPyV status

The positive status for MCPyV was observed in 38/56 (67.9%) patients. Fisher’s exact test showed that MCPyV status had no significant impact on detecting MCC nodal metastases. A similar distribution of SATB2, CKAE1/AE3, and synaptophysin among MCPyV-negative and MCPyV-positive cases was observed (Supplemental Fig. 6).

## Discussion

Microscopically identified regional lymph node involvement is the most critical independent unfavorable prognostic marker in MCC patients [16–18]. Santamaria-Barria et al. [19] revealed that one-third of early-stage patients (stages I and II) had micrometastases in sentinel lymph nodes (in 9 out of 27 patients (33%)) and 39% (7/18) of SLNB-negative patients developed recurrence. These clinical findings indicate that more effective histopathologic methods of detection of MCC nodal micrometastases are needed.

MCC metastasis detection requires the implementation of a uniform, highly reproducible protocol, which combines histologic examination followed by IHC stains according to a standardized algorithm [1, 20]. Our multi-institutional and comprehensive study analyses compare different IHC markers in detecting MCC metastases. We showed that a panel of three antibodies (SATB2, CKAE1/AE3, and synaptophysin) is the most helpful in routine pathological assessment. We recommend revising the MCC metastasis diagnostic guidelines since there is no one precise IHC protocol [20]. We highlight the importance of using optimal IHC supporting tools as finding even a single MCC metastatic cell changes the clinical staging and influence on treatment modification.

According to our results, the most promising antibody is SATB2, belonging to the SATB family proteins, which are fundamental regulators of gene expression and modifiers of chromatin structure [21]. We found that SATB2 was positive in all nodal metastases; nearly 90% of cases demonstrated positivity in ≥ 75% of tumoral cells. The stain interpretation was simple since its nuclear expression remained uniformly moderate to strong. In previous studies, SATB2 was described as an effective marker in the differential diagnosis of MCC and extracutaneous neuroendocrine carcinomas [10, 22, 23]. In skin, Fukuhara et al. [24] showed that normal Merkel cells present SATB2 reactivity, and their results confirmed the diagnostic utility of SATB2 in distinguishing MCC from other skin neoplasms. Kervarrec et al. [22] showed a high diagnostic accuracy of immunohistochemistry for SATB2 in detecting Merkel cell carcinoma versus extracutaneous neuroendocrine carcinomas with high specificity (98%) and a positive likelihood ratio of 36.6.

INSM1 is the second recently described protein tested in MCC detection [7]. Lilo et al. [11] showed that INSM1 stained > 75% of tumor nuclei in 89% of MCC, and it was influential in the distinction between MCC and other primary cutaneous neoplasms but not discriminating MCC from metastatic neuroendocrine carcinomas of extracutaneous origin. In our study, all metastases were INSM1 positive, with 73% cases with cut-off ≥ 75% stained nuclei. Surprisingly, there was no 100% INSM1 positive MCC, a significant limitation of its usage in SLNB evaluation; an unexpected observation was entirely negative mitotically active tumoral cells.

Cytokeratins have been recommended for MCC metastasis detection over the years [25, 26]. From various cytokeratins, CK20 with characteristic perinuclear dot-like positivity is the most specific [27–30]. Tetzlaff et al. [31] demonstrated the total percentage of CK20-positive vs. CK20-negative MCC as 87.4% vs. 12.6%, respectively. In our study, the IHC with the highest completely negative IHC was CK20 and comprised 7.8% of cases; moreover, 22.6% displayed positivity in < 25% of cells. Interpreting single dot-like reactions in lymph nodes is challenging; additionally, dendritic cells might express cytokeratins, but identifying spindle shape on closer inspection is supportive. We showed a superior diagnostic value of CKAE1/AE3 over the CK20, which was indicated by (1) a significantly higher percentage of tumoral cells with moderate to strong expression (83% vs. 59% of cases for CKAE1/AE3 vs. CK20, respectively), and (2) the total percentage of 100% positive cases, which constituted 32% for CKAE1/AE3 and 5% for CK20. We propose that wide-spectrum cytokeratin (CKAE1/AE3) should be maintained as the second antibody of choice (following the SATB2) for identifying MCC metastases.

Providing neuroendocrine origin of metastasis is required for MCC diagnosis confirmation. Kervarrec et al. [22] showed that NF is the most specific for MCC among all neuroendocrine markers. Most published NF images focus on dot-like reactions; our study proved that NF was uniformly expressed in 77.45% of cases presented as the scattered, dot-like reaction in 1–25% of MCC cells. In our opinion, this result and the fact there were no cases with high expression pattern (≥ 75% positive cells) disqualifies NF as a marker for SLNB assessment. By contrast, synaptophysin was a stable, positive IHC neuroendocrine marker in all cases, with no negative metastatic MCC lymph nodes. Although chromogranin A showed a higher number of 100% positive cases compared to synaptophysin (27.45% and 16.67%, respectively), it is not recommended for the histopathologic lymph node assessment because of its heterogeneity, presence of negative metastatic lymph nodes, and focally significant unspecified background of IHC reaction.

Importantly, a questionable aspect of all recommendations is the methodology of IHC interpretation. We used a highly reproducible 5-tier scoring system. On the contrary, implementing the H-score method in everyday practice is complex and characterized by the highest deviation among investigators [9, 32]. On the other hand, some simplified protocols are limited to reporting the lack of positive stain, or positive status was evaluated only on low microscopic magnification [33]. From our experience, we consider that it might lead to misinterpretation; the population of reactive lymphocytes may show weak to moderate SATB2 expression, which might be confused with MCC metastases. Careful comparison with the cell morphology at higher magnification (200 × or 400 ×) and/or the re-evaluation of the case with additional IHC (CKAE1/AE3 or synaptophysin) is helpful. In addition, a single MCC cell qualifies a node as metastatic, so there is an evident need to use higher magnification.

We acknowledge that there are some challenging pitfalls in MCC SLNB diagnostics. The nonspecific patterns observed for SATB2 and pan-keratin for detecting MCC would not be problematic in most cases. However, in the context of single metastatic cells or minute clusters, the interpretive difficulty could emerge; some pathologists might encounter a mildly enlarged and moderately SATB2-positive nucleus that is not clearly tumor vs. lymphoid or puncta of keratin that could be either a paranuclear dot or tangential cut of dendritic labeling. Such nonspecific patterns also increase the chance that single cells are missed as the pathologist examines across the lymph node whole sections. For this reason, pan-keratin has not been a stand-alone immunohistochemical marker for SLNB evaluation in MCC. By contrast, CK20 is highly specific, allowing for much greater confidence in detecting and diagnosing single-cell metastases. These practical considerations account for the frequent use of combined pan-keratin and CK20 stains for evaluating MCC SLNB, despite the known limitations of CK20. We recognize this weakness and recommend synaptophysin as a more specific marker, which has low but not zero cross-reactivity with normal populations in lymph nodes.

The strengths of our study are the following: (1) the comparison of well-established IHC markers (CKAE1/AE3, CK20, synaptophysin, chromogranin A, and NF) with recently available new antibodies (SATB2 and INSM1) for MCC metastasis identification; (2) the high number of evaluated cases; (3) providing assessment intertumoral heterogeneity in multiple metastases; (4) emphasis on the practical aspects by implementing a user-friendly, semiquantitative, reproducible scoring system that pathologists could apply in routine diagnostics. We are aware that the limitations include (1) tissue microarray instead of whole slide evaluation and (2) lack of comparison with primary and metastasic MCC. To minimize weaknesses, we used at least two samples (cores) from one metastasis and analyzed the intratumoral heterogeneity whenever possible (multiple metastases cases). According to the literature review, the immunoprofile of MCC primary showed strong convergence with metastatic [31].

In conclusion, we have shown that SATB2 would be helpful in MCC as the first-line marker in the SLNB/LNB histopathologic assessment. We also suggest including CKAE1/AE3 and synaptophysin, which should be performed sequentially after SATB2. These are widely available antibodies that can be easily implemented; the pathological, semiquantitative interpretation is also reproducible and not particularly time-demanding. Since no specific IHC marker exists, the neuroendocrine metastases originating from other sites should be excluded.

### Supplementary Information

Below is the link to the electronic supplementary material.

Supplementary file1 (JPG 409 KB) SATB2 expression in reactive lymphoid tissue (tonsil)

Supplementary file2 (PNG 236 KB) Distribution of IHC markers in 102 metastatic MCC lymph nodes. The diagram shows the distribution of node occupancy for separate markers. Each bar represents a different protein and the values represent the number of nodes (out of 102 examined nodes) for which the fraction of positive cells is in the color-coded range

Supplementary file3 (PNG 489 KB) Diagram shows the pairwise correlations and relationships between the investigated markers. Each row and column regards one protein (name located at the top and on the right). On the left and bottom, there are values describing the positive cell fraction range. The diagonal shows the distribution of results for individual proteins (bar labels indicate the number and percentage share of nodes with a given rating). Above the diagonal, the values of the Spearman's correlation coefficient and their associated p-values are presented (each result is for a row-column protein pair). Whereas, below the diagonal, the relationships are shown numerically - each number informs about common occurrences of given ratings. For example, the correlation coefficient between pan CK and CK20 is 0.42 (*p* = 0), and 21 nodes simultaneously received a rating of 4 for the CKAE1/AE3 protein and 3 for the CK20 protein

Supplementary file4 (PNG 142 KB) Measurement of heterogeneity by Leti index. This index was calculated for each patient with more than one node scored, separately for each protein. The results are presented on boxplots for each protein, along with the signature of the patients for whom the deviations were the greatest

Supplementary file5 (PNG 231 KB) Diagram shows the heterogeneity of the results for patients with more than one node examined. Each panel covers the results for one selected protein. Patient IDs are on the Y-axis and lymph node IDs are on the X-axis. The positive cell fraction in a given node has been color-coded using the same colors as in the other plots

Supplementary file6 (PNG 259 KB) Fisher's exact test showed that MCPyV status has no significant impact on detecting MCC nodal metastases; the similar distribution of SATB2, CKAE1/AE3 and synaptophysin among MCPyV-negative and MCPyV-positive cases was observed

## Data Availability

The datasets used and/or analyzed in this study are available from the corresponding author upon reasonable request.
