# Supplementary figures and images for: SATB2, CKAE1/AE3, and synaptophysin as a sensitive immunohistochemical panel for the detection of lymph node metastases of Merkel cell carcinoma
Source: Virchows Arch. 2023 Dec 8;484(4):629–36. doi: 10.1007/s00428-023-03691-7 (PMC11062961; doi:10.1007/s00428-023-03691-7)

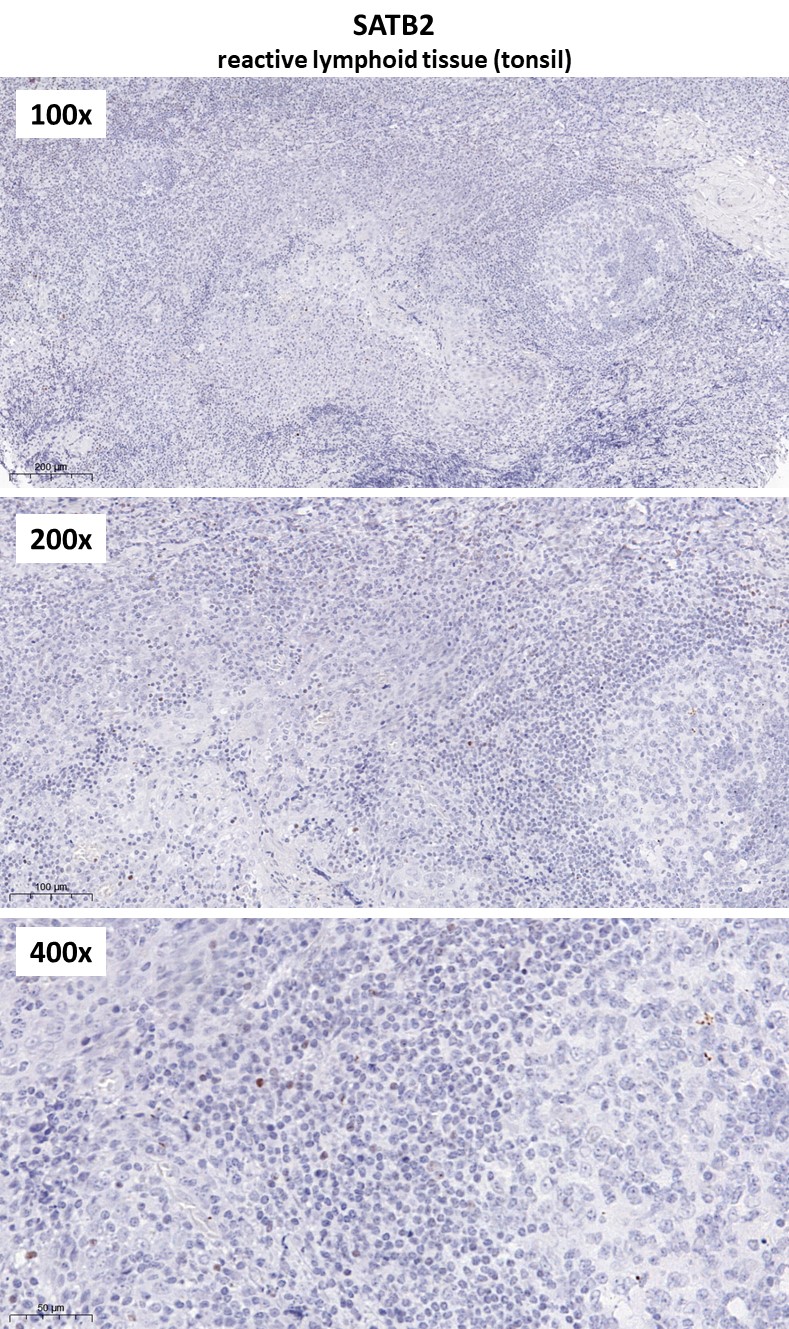

Supplement: Supplementary file 1 — Supplementary file1 (JPG 409 KB) SATB2 expression in reactive lymphoid tissue (tonsil) [file 428_2023_3691_MOESM1_ESM.jpg]

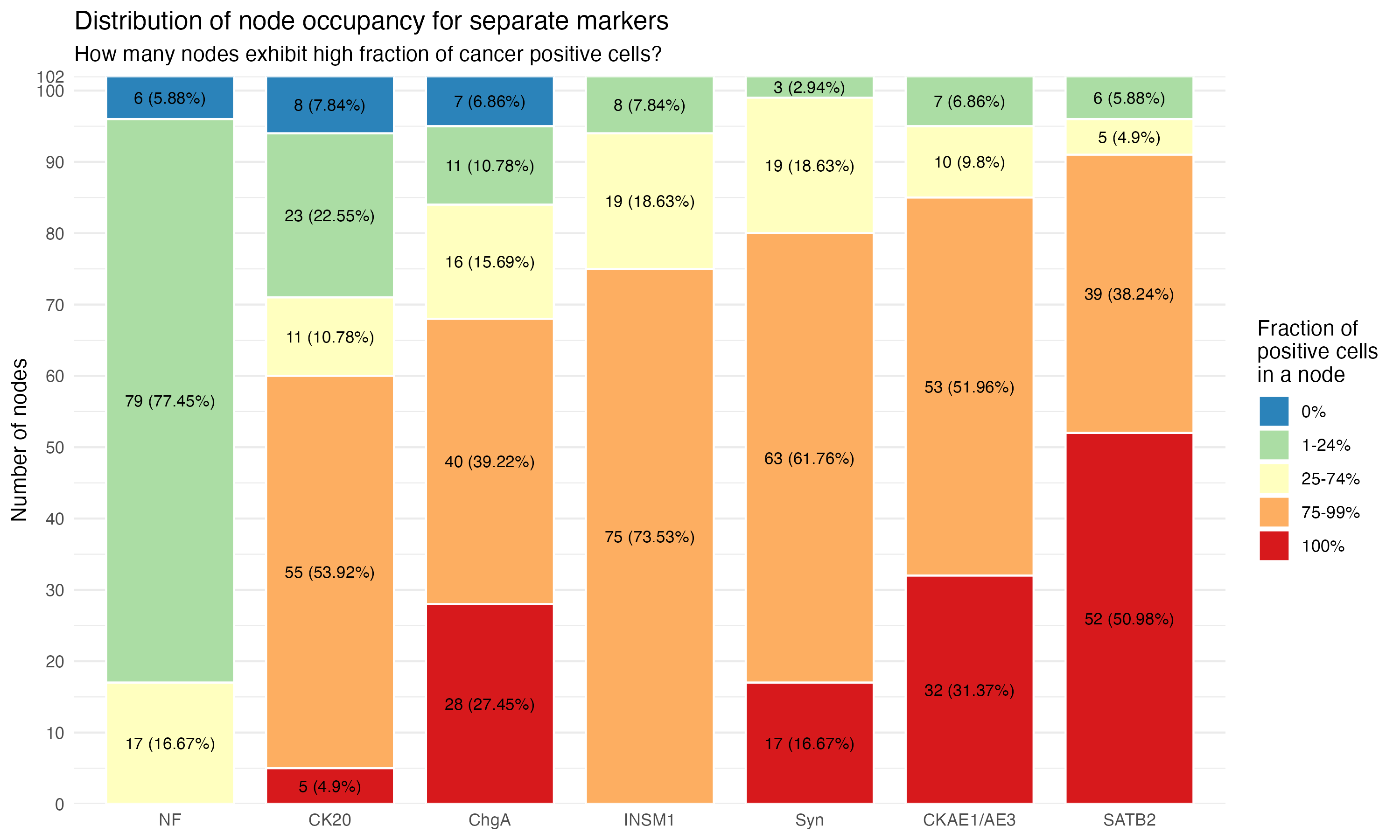

Supplement: Supplementary file 2 — Supplementary file2 (PNG 236 KB) Distribution of IHC markers in 102 metastatic MCC lymph nodes. The diagram shows the distribution of node occupancy for separate markers. Each bar represents a different protein and the values represent the number of nodes (out of 102 examined nodes) for which the fraction of positive cells is in the color-coded range [file 428_2023_3691_MOESM2_ESM.png]

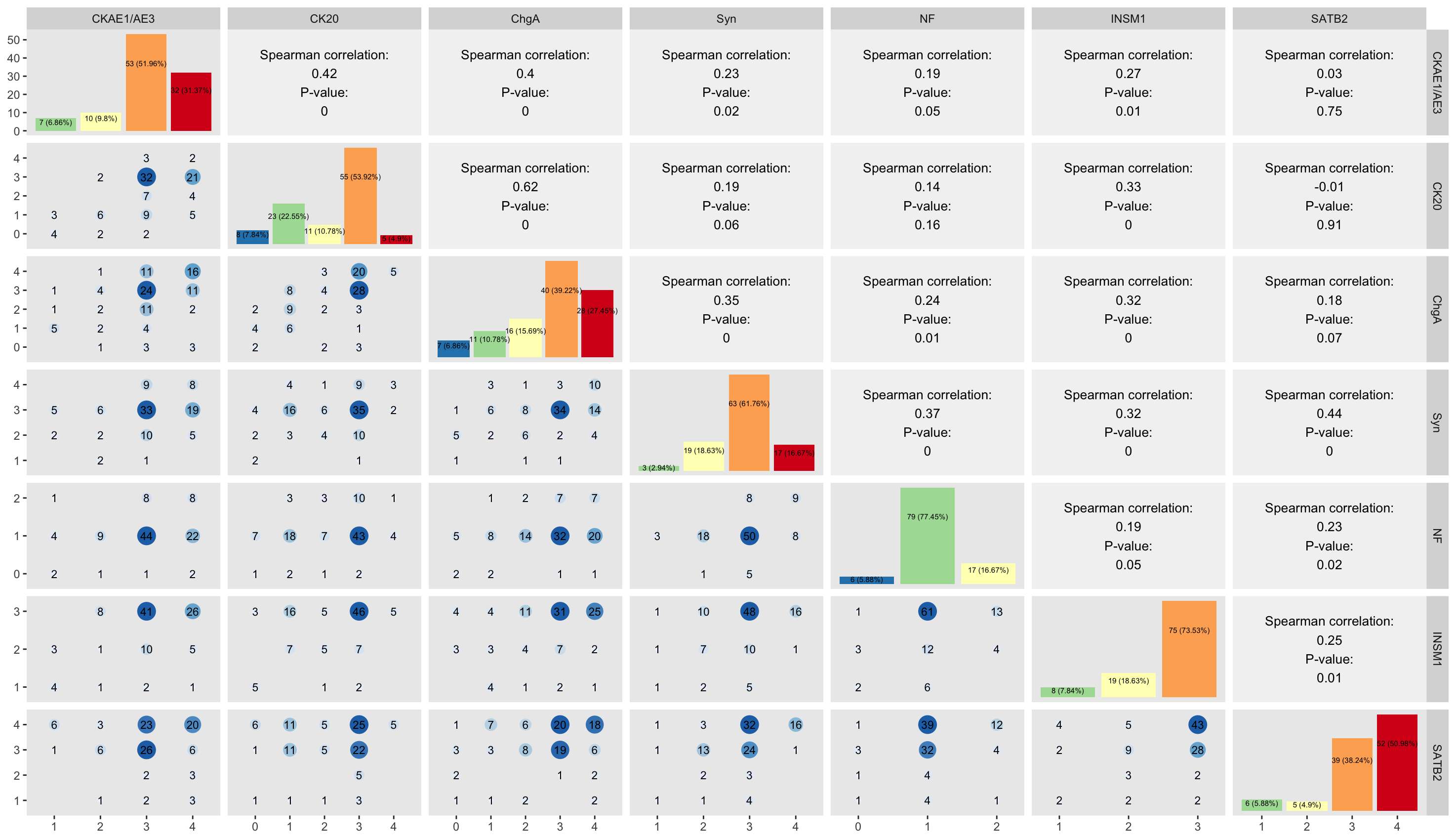

Supplement: Supplementary file 3 — Supplementary file3 (PNG 489 KB) Diagram shows the pairwise correlations and relationships between the investigated markers. Each row and column regards one protein (name located at the top and on the right). On the left and bottom, there are values describing the positive cell fraction range. The diagonal shows the distribution of results for individual proteins (bar labels indicate the number and percentage share of nodes with a given rating). Above the diagonal, the values of the Spearman's correlation coefficient and their associated p-values are presented (each result is for a row-column protein pair). Whereas, below the diagonal, the relationships are shown numerically - each number informs about common occurrences of given ratings. For example, the correlation coefficient between pan CK and CK20 is 0.42 (p = 0), and 21 nodes simultaneously received a rating of 4 for the CKAE1/AE3 protein and 3 for the CK20 protein [file 428_2023_3691_MOESM3_ESM.png]

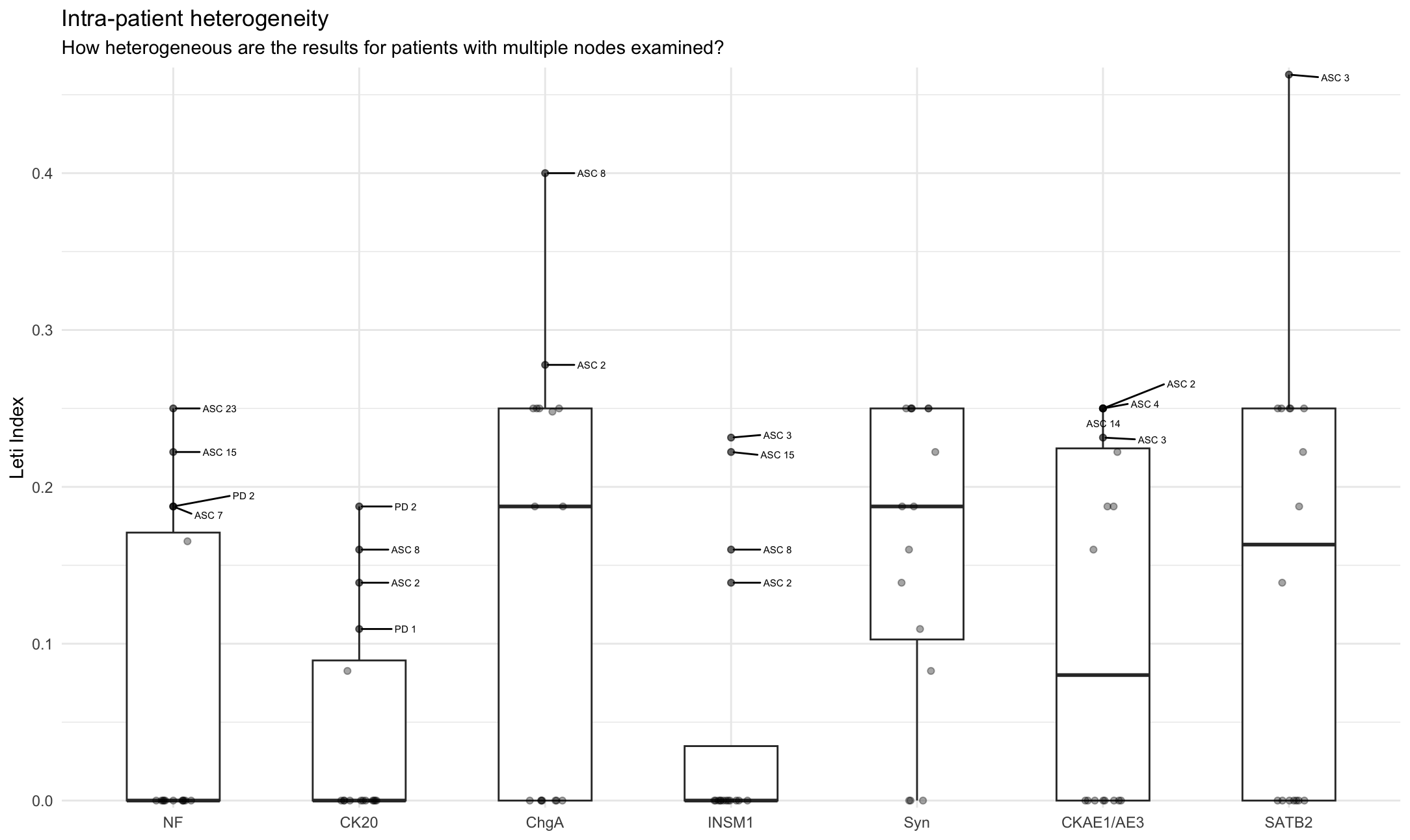

Supplement: Supplementary file 4 — Supplementary file4 (PNG 142 KB) Measurement of heterogeneity by Leti index. This index was calculated for each patient with more than one node scored, separately for each protein. The results are presented on boxplots for each protein, along with the signature of the patients for whom the deviations were the greatest [file 428_2023_3691_MOESM4_ESM.png]

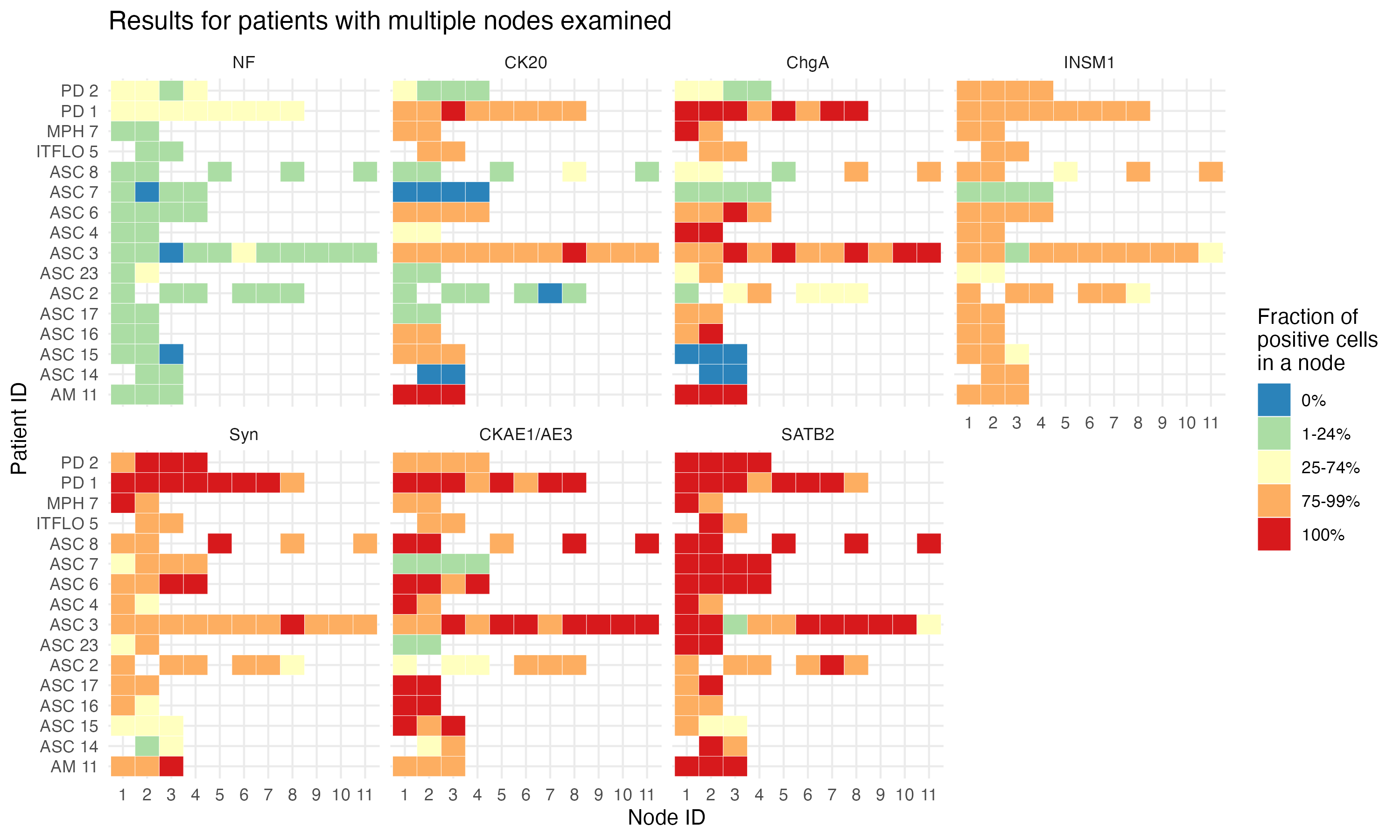

Supplement: Supplementary file 5 — Supplementary file5 (PNG 231 KB) Diagram shows the heterogeneity of the results for patients with more than one node examined. Each panel covers the results for one selected protein. Patient IDs are on the Y-axis and lymph node IDs are on the X-axis. The positive cell fraction in a given node has been color-coded using the same colors as in the other plots [file 428_2023_3691_MOESM5_ESM.png]

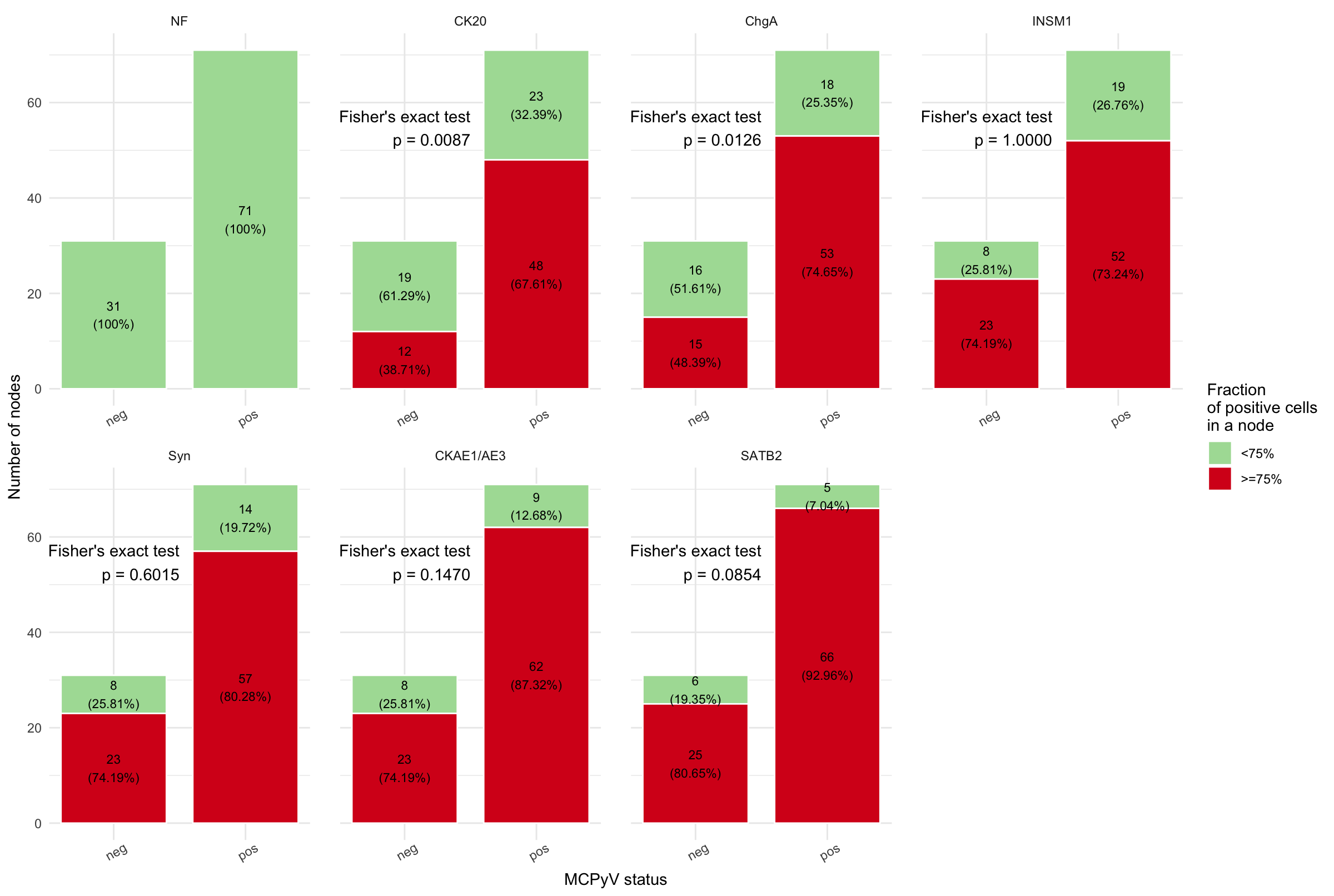

Supplement: Supplementary file 6 — Supplementary file6 (PNG 259 KB) Fisher's exact test showed that MCPyV status has no significant impact on detecting MCC nodal metastases; the similar distribution of SATB2, CKAE1/AE3 and synaptophysin among MCPyV-negative and MCPyV-positive cases was observed [file 428_2023_3691_MOESM6_ESM.png]
